# Methodological quality of systematic reviews of animal studies: a survey of reviews of basic research

**DOI:** 10.1186/1471-2288-6-10

**Published:** 2006-03-13

**Authors:** Luciano E Mignini, Khalid S Khan

**Affiliations:** 1 Centro Rosarino de Estudios Perinatales, Rosario Argentina; 2 Department of Obstetrics & Gynaecology, Birmingham Women's Health Care NHS Trust, Birmingham, UK

## Abstract

**Background:**

Systematic reviews can serve as a tool in translation of basic life sciences research from laboratory to human research and healthcare. The extent to which reviews of animal research are systematic and unbiased is not known.

**Methods:**

We searched, without language restrictions, Medline, Embase, bibliographies of known reviews (1996–2004) and contacted experts to identify citations of reviews of basic science literature which, as a minimum, performed search of a publicly available resource. From these we identified reviews of animal studies where laboratory variables were measured or where treatments were administered to live animals to examine their effects, and compared them with reviews of bench studies in which human or animal tissues, cell systems or organ preparations were examined in laboratories to better understand mechanisms of diseases.

**Results:**

Systematic reviews of animal studies often lacked methodological features such as specification of a testable hypothesis (9/30, 30%); literature search without language restriction (8/30, 26.6%); assessment of publication bias (5/30, 16.6%), study validity (15/30, 50%) and heterogeneity (10/30, 33.3%); and meta-analysis for quantitative synthesis (12/30, 40%). Compared to reviews of bench studies, they were less prone to bias as they specified the question (96.6% vs. 80%, p = 0.04), searched multiple databases (60% vs. 26.6%, p = 0.01), assessed study quality (50% vs. 20%, p = 0.01), and explored heterogeneity (33.3% vs. 2.2%, p = 0.001) more often.

**Conclusion:**

There seems to be a gradient of frequency of methodological weaknesses among reviews: Attempted systematic reviews of whole animal research tend to be better than those of bench studies, though compared to systematic reviews of human clinical trials they are apparently poorer. There is a need for rigour when reviewing animal research.

## Background

In the development of new health technologies, it is widely held that drugs or procedures should first be assessed in animal models before proceeding to clinical trials in humans[1]. High quality systematic reviews provide unbiased overviews of the available evidence[2]. There have been calls for application of this approach in basic research, particularly in animal research[3], to better understand biological plausibility [4-7] and to translate findings of basic research to the bedside[8]. Cumulative assessment of emerging evidence in animal research can help rationalize human clinical trials[9]. The idea that these experiments impact future human studies is well recognised, but lack of systematic review of this evidence can lead to a sort of research bias that has seldom been previously considered explicitly. The link (or lack of appreciation of a link) between animal and human studies is illustrated by the case of nimodipine in focal cerebral ischemia; it has become clear from systematic review of animal experiments that there was no convincing evidence to substantiate the decision to perform trials with nimodipine in humans[10]. Because the initial animal studies were not evaluated systematically; human trials of nimodipine proceeded at significant cost and potential human risk despite a lack of clear scientific rationale. The extent and the quality of systematic reviews of animal studies is unknown. The aim of this study was to assess the methodological features of such systematic reviews.

## Methods

**Figure 1:**
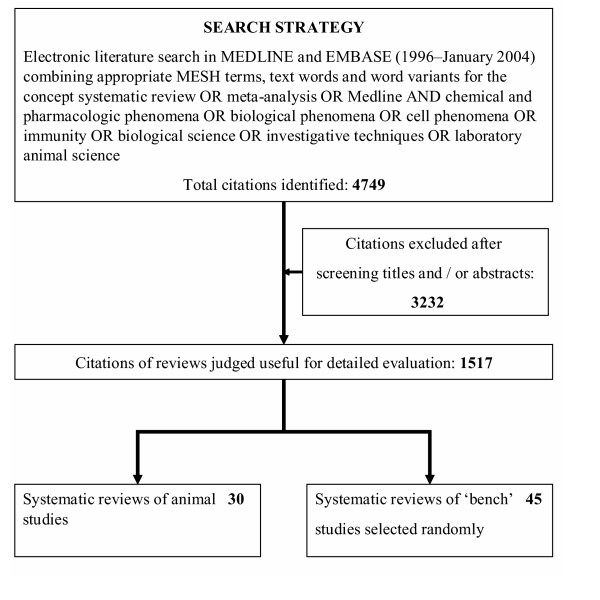
Search strategy and selection process for identifying reviews of animal and bench studies.

We searched Medline and Embase (1996–2004) using a search term combination (Figure 1) carefully developed with input from expert librarians (LIS-MEDICAL@JISCMAIL.AC.UK), as there is no standard approach to indexing citations of systematic reviews of animal or basic research in life sciences. To identify reviews not captured by our electronic database search, we examined bibliographies of known reviews and contacted research experts through an e-mail list discussion group (EVIDENCE-BASED-HEALTH@JISCMAIL.AC.UK). References listed in primary studies and reviews were scrutinized to further identify other studies not captured by electronic searches. Where possible, authors of relevant studies and several experts in the field were contacted in an attempt to identify additional references, unpublished and/or ongoing studies, or unpublished data.

Studies were selected in two stages. Initially, one of the authors (LM) independently scrutinized the electronic searches and obtained the manuscripts of all citations that appeared to meet our predefined selection criteria. Inclusion or exclusion decisions were made only after examination of the manuscripts. Where multiple analyses from a single study were published separately, we used the latest publication for results, supplementing information on study characteristics and quality from earlier publications if necessary. Two authors (LM and KSK) independently assessed all English language manuscripts, while articles in other languages were assessed by people familiar with the language under the supervision of LM. Disagreements about inclusion or exclusion were resolved by consensus.

We selected reviews of animal studies which, at a minimum, performed search of a publicly available resource. We defined animal studies as those in which laboratory variables were measured or where treatments were administered to live animals to examine their effects. We compared their findings with those of reviews of bench studies, defined as research in which human or animal tissues, cell systems or organ preparations were examined in laboratories to better understand disease mechanisms. We used bench studies for comparison because they fall within the category of laboratory studies in life sciences along with animal studies. We took care to ensure that reviews of animal and bench studies were mutually exclusive and we planned separate evaluations in case of overlap. We selected all reviews of animal studies (n = 30). For bench studies we created lists of citations for each year and serially numbered them. We used a random numbers table to select two citations (or one citation where this was not possible) of reviews of bench studies for each review of animal studies from the same publication year without language restrictions (Figure 1). This generated a sample of reviews of bench studies for comparison (n = 45). Our sample allowed us to determine a 50% difference (35% vs 70%) in key methodological features between the two review types at an α of 5% and β of 20%.

**Figure 2:**
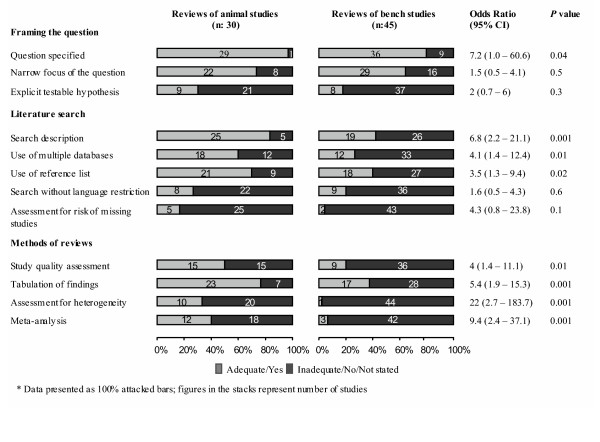
Features of systematic reviews included.

Our review protocol was designed to examine the methodological quality of reviews of animal studies, using recommended methods for conducting such systematic reviews [11-16]. These methodological guides resemble those available for evaluation of systematic reviews of human studies [17-24]. Our checklist consisted of 12 items divided into three domains concerning the review question, the literature search and the review methods (Figure 2). These items assessed the risk of errors and bias in the review process. A 'good' quality item was one where there was a clear description in the report of compliance with the items, whereas a 'bad' quality item either did not comply with or did not report sufficient details to assess the item. Not all items related to bias; some related to explicitness of reporting that affords scientific transparency in a review. One of us (LM) extracted data from the identified papers and a second reviewer (KSK) independently checked them for errors. We did not undertake formal agreement studies; however, we report all our findings in the data tables explicitly so an interested reader can independently examine our data extraction for accuracy.

Group comparisons were made using Chi-square or Fisher's exact tests for differences in proportions/percentages. The level of significance was set at α = 0.05 but exact P values are provided so readers could use a more stringent threshold if they wished. Odds ratios and their 95% confidence intervals (CI) were computed.

## Results

From 4749 citations initially identified, 1517 were considered potentially relevant and their full manuscripts were evaluated. Among these, there were only 30 (1.9% of 1517) reviews of animal studies that met our selection criteria (Figure 1). The reviews summarised studies of animals including cat, cow, horse, dog, mouse, nonhuman primate, rabbit, rat, sheep and swine amongst others. The ranges of topics included cardiology, dentistry, gynaecology,  immunology, neonatology, obstetrics, oncology, toxicology and urology  amongst others as shown in Appendix 1 (Additional File 1).

Figure 2 shows that reviews of animals studies often lacked methodological features such as specification of a testable hypothesis (9/30, 30%); literature search without language restriction (8/30, 26.6%); assessment of publication bias (5/30, 16.6%), study quality (15/30, 50%) and heterogeneity (10/30, 33.3%); and meta-analysis for quantitative synthesis (12/30, 40%). However, compared to reviews of bench studies, they were less prone to bias as they specified the question (96.6% vs. 80%, p = 0.04), searched multiple databases (60% vs. 26.6%, p = 0.01), assessed study quality (50% vs. 20%, p = 0.01), and explored heterogeneity between studies (33.3% vs. 2.2%, p = 0.001) more often (Figure 2).

## Discussion

The principle of systematically reviewing animal and bench research is well established. However, even with a lenient definition for systematic reviews, such reports of animal studies were relatively infrequent considering the large amount of research funding in this field. These reviews were generally poor in their reporting of methodological quality features. Although they had better compliance with methodological features than reviews of bench studies, they were deficient compared to reviews of clinical trials [25-27]. For example, compared with Cochrane reviews of clinical trials[28], reviews of animal studies have used searches without languages restriction [8/30; 26.6% vs. 36/36; 100%] (p < 0.001), assessment of the quality of the studies included [15/30; 50% vs. 36/36; 100%] (p < 0.001) and data synthesis methods [12/30; 40% vs. 33/36; 92%] (p < 0.001) less frequently.

The validity of our findings is inherent in the quality of our study. We complied with a rigorous *a priori *protocol which reduces the risk of bias due to hindsight. There are a few potential limitations of our work. For example, as no sensitive search filters exist for animal or bench studies, our search may have failed to capture all eligible reviews. Another limitation, the lack of duplicate independent assessments, could introduce errors in data extraction. We did perform double checks although these were not blinded or independent. As we report all our findings transparently, an interested reader can independently examine the accuracy of our data extraction. Yet another perceived deficiency could be the choice of reviews of bench studies as our comparison group. Prior to our study, little has been known about the methodological quality of reviews of bench studies, so some might argue that reviews of human studies could have served as a better comparator. It is the case that methodological quality of reviews of human clinical trials has been extensively evaluated in a variety of areas; however, the same cannot be said of reviews of human observational studies. Thus it is unclear which subgroup of human studies could serve as an appropriate comparator. If only reviews of clinical trials are chosen for comparison, reviews of animal studies would have an unfair disadvantage mainly because the tradition of reviewing research based on randomised trials is firmly established. We believe the reviews of bench research serve as the most appropriate comparator for examining quality of reviews of animal research as both belong to the basic biomedical research domain. We do provide an indirect comparison against published evaluations of quality of reviews of clinical trials (above) for reference.

From our study, a number of lessons have emerged for reviewers of animal research. When interrogating databases for reviews of animal or basic research searches could be restricted if the majority of the work undertaken is kept confidential[29]. In this situation, systematic reviews are likely to be flawed, particularly if clear evidence of publication bias can be demonstrated. The proportion of the work that gets published in a form that is available to the public (rather than just being available to industry and the regulatory authorities) is unknown. However, assessments for risk of missing studies scarcely featured in the reviews we assessed. Special efforts (contact with experts, laboratories and other related research associations) will be needed to retrieve unpublished data. This is one of most important challenges for reviewers of animal studies.

Validity or quality of studies included in a review is a key issue in avoiding bias in biological and laboratory methods. Despite its importance this issue was often not assessed in the reviews we studied, increasing the risk of drawing erroneous inferences. In our study, the proportion of reviews that included meta-analysis was small, but the actual need for use of this statistical technique is unknown. Disturbingly, we found that data synthesis among the reviews included in our study usually ignored methods to assess heterogeneity such that the suitability of combining results in meta-analysis could not be evaluated. Our study reflects the poor state of reviews in animal and basic life sciences research.

We conclude that attempted systematic reviews of bench studies have a higher proportion of methodological weaknesses than those of whole animal studies, though apparently the latter are not as good as systematic reviews of human clinical trials for comparable standards. Given the importance of animal and basic research for scientific and clinical practice, there is a need for greater awareness about assembly and appraisal of the relevant literature comprehensively and systematically in reviews [30-34]. Indeed, it can be argued that the need is even greater in basic research since these results influence the decision as to which clinical studies are undertaken. We suggest that systematic reviews of animal and bench studies should be an essential prerequisite before results are further tested in human clinical trials.

## Competing interests

The author(s) declare that they have no competing interests.

## Authors' contributions

LM conducted the searches. LM and KSK extracted, analyzed and interpreted the data. Both authors drafted and approved the final version of the manuscript.

## Pre-publication history

The pre-publication history for this paper can be accessed here:



## Supplementary Material

Additional File 1 Click here for file
