# Supplementary material for: Methodological quality of systematic reviews of animal studies: a survey of reviews of basic research
Source: BMC Med Res Methodol. 2006 Mar 13;6:10. doi: 10.1186/1471-2288-6-10 (PMC1435907; doi:10.1186/1471-2288-6-10)
Supplement: Additional File 1 [file 1471-2288-6-10-S1.doc]

| **Appendix 1: Features of systematic reviews included** | | | | | | | | | | | | | | | |
| --- | --- | --- | --- | --- | --- | --- | --- | --- | --- | --- | --- | --- | --- | --- | --- |
|  | **Topic and question** | | | | | | | **Literature search** | | | | **Methods of reviews** | | | |
| Author, Year | Topic | Question specified  (Focus  of the question) | | Nature of question:  Mechanisms of | | Explicit testable hypothesis | Search description | Database searched | Use of reference list | Language restriction | Assessment of risk of missing studies | Study quality assessment | Tabulation of the findings | Assessment for heterogeneity | Meta-analysis |
|  |  |  |  | disease | action |  |  |  |  |  |  |  |  |  |  |
| **A. Systematic reviews of animal studies** | | | | | | | | | | | | | | | |
| Borreli, 2003^1^ | Pharmacological effects of Cimicifuga racemosa in dogs, rats, mice | Yes  (Broad) | | No | Yes | No | Adequate | Medline, Embase, Cochrane Library, CISCOM (Research Council for Complementary Medicine), Cimicifuga racemosa manufacturers | Yes | No | No | No | Yes | No | No |
| Bayley B, 2003^2^ | Efficacy of glucagon in the treatment of b-blocker and calcium channel blocker overdoses in Dogs, rats, rabbits | Yes  (Narrow) | | No | Yes | No | Adequate | Medline, Embase. Cochrane, Toxline | Yes | No | Not stated | Yes | Yes | No | No |
| Corpet D, 2003^3^ | Chemoprevention of colon cancer in rats | Yes  (Narrow) | | No | Yes | Yes | Adequate | Medline, Current Contents Life Science, American Association for Cancer Research, Carcinogenesis, Cancer letters | Yes | No | No | Yes | Yes | No | No |
| Dirx M, 2003^4^ | Effects of energy restriction on the risk of spontaneous mammary tumors in mice | Yes  (Narrow) | | Yes | No | No | Adequate | Medline, Current contents | Yes | Yes | No | Yes | Yes | Yes | Yes |
| Holemans K, 2003^5^ | Effects of fetal growth restriction of offspring in rats | Yes  (Narrow) | | Yes | No | No | Inadequate | Medline | Not  stated | No | Not stated | No | No | No | No |
| Lee D, 2003^6^ | Effect of endothelin receptor blockers in experimental heart failure in rats | Yes  (Narrow) | | No | Yes | Yes | Inadequate | Medline, Embase | Yes | Yes | No | Yes | Yes | Yes | Yes |
| MapstoneJ, 2003^7^ | Fluid resucitation strategies in uncontrolled hemorrhage in Swine, sheep, rats | Yes  (Broad) | | No | No | Yes | Adequate | Medline, Embase | Yes | No | Not stated | Yes | Yes | Yes | Yes |
| Meune C, 2003^8^ | Risk and benefits of NSAIDs including aspirin in miocarditis in mice | Yes  (Narrow) | | No | Yes | No | Adequate | Medline | Yes | No | Not stated | No | Yes | No | No |
| Quadrilatero J, 2003^9^ | Physical activity and colon cancer | Yes  (Broad) | | Yes | No | Yes | Adequate | Medline | Yes | No | Yes | Yes | Yes | No | Yes |
| Rehman J, 2003^10^ | Tissue chemoablation in rats and mouse. | Yes  (Broad) | | No | Yes | No | Inadequate | Medline | Not  stated | No | Not stated | No | No | No | No |
| Ren Y, 2003^11^ | Optimal force or range of forces for orthodontic tooth movement in Cat, dog, monkey, rat, rabbit and guinea pig. | Yes  (Narrow) | | Yes | No | No | Adequate | Medline | Yes | No | Not stated | Yes | Yes | No | No |
| Aghjafari F, 2002^12^ | Effects of repeated doses of antenatal corticorteroids on lung and brain function and on growth restriction in Sheep, monkeys, rabbits, mice | Yes  (Narrow) | | No | Yes | No | Adequate | Medline, Embase | Not  stated | No | Yes | No | Yes | No | No |
| Fox C, 2002^13^ | Effects of phytic acid as an anti-neoplastic agent in Rats, mice, elephants. | Yes  (Broad) | | No | Yes | No | Adequate | Medline | Not  stated | No | Not stated | No | No | No | No |
| Kilborn S, 2002^14^ | Growth palte closure copmpared with age at sexual maturity and lifespan in Mouse, rat, rabbit, dog, cat, sheep, cow, horse, nonhuman primate. | Yes  (Narrow) | | No | No | No | Adequate | Medline, American College of Laboratory Medicine and Veterinary Clinics of North America | Yes | No | Not stated | No | Yes | No | No |
| Klinge B, 2002^15^ | Effect of anti-infective therapy in the treatment of prei-implantitis in Dogs, monkeys, pigs. | Yes  (Narrow) | | No | Yes | No | Adequate | Medline and hand searching of related journals | Yes | No | No | Yes | Yes | No | No |
| Lucas C, 2002^16^ | Wound healing in cell by low laser therapy in Rats, pigs, mice, pigs, rabbits. | Yes  (Narrow) | | No | Yes | Yes | Adequate | Medline, embase, SPIE (International Society for Optical Engineering) | Yes | No | Yes | Yes | Yes | Yes | Yes |
| Blackman K, 2001^17^ | Effect of plasma alkalinization for triciclic antidepressant toxicity in Dogs, swines. | Yes  (Narrow) | | No | Yes | No | Adequate | Medline | Yes | No | No | No | Yes | No | No |
| Boisser J, 2001^18^ | Relation between worn burden and male proportion in Shistosoma mansoni in Rats, pigs, mice, hamsters, monkeys. | Yes  (Narrow) | | Yes | No | Yes | Inadequate | Medline, local database | Not  stated | No | Not stated | Yes | Yes | No | Yes |
| Collins J, 2001^19^ | Effect of formaldehyde on adverse pregnancy outcome in Rats, dogs, mice, hamsters, rabbits | Yes  (Narrow) | | No | Yes | No | Adequate | Medline, toxline | Not  stated | Yes | No | Yes | Yes | Yes | Yes |
| Horn J, 2001^20^ | Effect of nimodipine in focal cerebral ischemia in Rats, rabbits, cats, mongolian gerbilis. | Yes  (Narrow) | | No | Yes | No | Adequate | Medline, Embase | Yes | No | Yes (english, german, french) | Yes | Yes | Yes | Yes |
| Rantala H, 2001^21^ | The role of prostaglandins and their synthethase inhibitars with respect to febrile seizures in Rat mouse, baboon | Yes  (Narrow) | | No | Yes | No | Adequate | Medline | Yes | No | Yes | No | Yes | No | No |
| Sherer D, 2001^22^ | Angiogenesis of the endometrium | Yes  (Broad) | | Yes | No | No | Adequate | Medline, Current Contents, and the Index Medicus | Yes | No | Yes | No | No | No | No |
| Fishbain D, 2000^23^ | Effect of antidepressant in pain relief in mice and mouse | Yes  (Narrow) | | No | Yes | Yes | Adequate | Medline, Science Citation index, Psych Info, National Library of Medicine (Physician Data Query) | Yes | Yes | No | No | No | No | No |
| Glatt S, 2000^24^ | Effect of prenatal cocaine exposure on dopamine system development in rats | Yes  (Narrow) | | Yes | Yes | No | Adequate | Medline | Yes | No | Yes | Yes | Yes | Yes | Yes |
| Nava-Ocampo A, 2000^25^ | Effect of pre-synaptic blockade of glutamate on neuroprotection | Yes  (Narrow) | | No | Yes | Yes | Adequate | Medline | Not  stated | No | Yes | No | Yes | Yes | Yes |
| Abulafia O, 1999^26^ | Angiogenesis of the endometrium | Yes  (Narrow) | | Yes | No | No | Adequate | Medline, Current Contents, and the Index Medicus | Not  stated | No | Not stated | Not  stated | No | No | No |
| Vickers A, 1999^27^ | Independent replication of pre-clinical research in homoeopathy in Rats, mice, chickens, albino guinea pigs, frogs, rabbits. | No  (Broad) | | No | No | No | Adequate | Medline, Embase, Cochrane controlled trials register, CISCOM (Research Council for Complementary Medicine), Phycoinfo, Cinahl. | Yes | Yes | Not stated | Yes | Yes | No | No |
| Kelley G, 1996^28^ | Effect of mechanical overload on sckeletal muscle fiber in Quail, Chicken, rats, cats, mice. | Yes  (Narrow) | | Yes | No | No | Inadequate | Medline | Yes | No | Yes | Yes | Yes | Yes | Yes |
| Laviano A, 1996 | Treatment of malnutrition in cancer patients^29^ | Yes  (Broad) | | Yes | No | No | Adequate | Medline | Not  stated | No | Yes | Not  stated | No | No | No |
| Perna A, 1996^30^ | Abnormal permeability to proteins and glomerular lesions in rats. | Yes  (Narrow) | | Yes | No | Yes | Adequate | Medline | Yes | No | Yes | No | Yes | Yes | Yes |
| **B. Systematic reviews of ‘bench’ studies** | | | | | | | | | | | | | | | |
| Abraham E, 2003^31^ | Neutrophils and acute lung injury | | Yes  (Narrow) | Yes | No | No | Inadequate | Medline | Not  stated | No | Not stated | Not  stated | No | No | No |
| Alam M, 2003^32^ | Effects of nonablative laser Therapy | | Yes  (Narrow) | No | Yes | No | Inadequate | Medline | Yes | No | Yes | Not  stated | No | No | No |
| Al-Sukhun S, 2003^33^ | Biology of advance bladder cancer | | No  (Broad) | Yes | No | No | Inadequate | Medline | Not  stated | No | Not stated | Not  stated | No | No | No |
| Bellanti J, 2003^34^ | Human immune system and its interaction with external environmental factors | | Yes  (Broad) | Yes | No | Yes | Inadequate | Medline | Not  stated | No | Yes | Not  stated | No | No | No |
| Creteur J, 2003^35^ | Cell-free hemoglobin solutions | | No  (Broad) | No | Yes | No | Inadequate | Medline | Yes | No | Not stated | Not  stated | No | No | No |
| Debattista J, 2003^36^ | Immunopathogenesis of chlamydia trachomatis infections in women | | Yes  (Narrow) | Yes | No | No | Adequate | Medline | Not  stated | No | Not stated | Not  stated | No | No | No |
| Duggan B, 2003^37^ | Molecular Profiling of Tumor Cells in Prostate and Bladder Cancer | | Yes  (Narrow) | Yes | No | No | Inadequate | Medline | Not  stated | No | Not stated | Not  stated | Yes | No | No |
| Fenster B,2003^38^ | Endothelial dysfunction: clinical strategies for treating oxidant stress | | No  (Broad) | Yes | Yes | No | Inadequate | Medline | Not  stated | No | Yes | Not  stated | Yes | No | No |
| Garcea G, 2003^39^ | Molecular biomarkers of colorectal carcinogenesis | | Yes  (Narrow) | Yes | No | No | Adequate | Medline, Web Science | Not  stated | No | Not stated | Not  stated | No | No | No |
| Kanayama N, 2003^40^ | Trophoblastic injury and pre-eclampsia | | Yes  (Narrow) | Yes | No | Yes | Adequate | Medline | Yes | No | Yes | Not  stated | No | No | No |
| Koornstra J, 2003^41^ | Apoptosis during the development of colorectal cancer | | Yes  (Narrow) | Yes | No | No | Adequate | Medline | Yes | No | Yes | Yes | Yes | No | No |
| Lyall M, 2003^42^ | Immunology of chronic fatigue syndrome | | Yes  (Narrow) | Yes | No | No | Adequate | Medline, Embase, Phycoinfo | Yes | Yes | Not stated | Yes | Yes | No | No |
| Mendez J, 2003^43^ | Advanced glycation end product and chronic complications of diabetes mellitus | | Yes  (Narrow) | Yes | No | No | Inadequate | Medline | Not  stated | No | Not stated | Not  stated | No | No | No |
| Norbury R, 2003^44^ | The neuroprotective effects of estrogen on the aging brain | | Yes  (Broad) | Yes | No | No | Inadequate | Medline | Yes | No | Not stated | Not  stated | No | No | No |
| Poon R, 2003^45^ | Angiogenesis in Gastrointestinal Cancers | | Yes  (Broad) | Yes | No | No | Adequate | Medline | Not  stated | No | Not stated | Yes | Yes | No | Yes |
| Reijnen M, 2003^46^ | Pathophysiology of intra-abdominal adhesion and abscess formation, and the effect of hyaluronan | | Yes  (Narrow) | Yes | Yes | No | Inadequate | Meline | Not  stated | No | Not stated | Not  stated | No | No | No |
| Szabó C, 2003^47^ | Role of flagellin in the pathogenesis of shock and acute respiratory distress syndrome: Therapeutic opportunities | | No  (Narrow) | Yes | No | No | Inadequate | Medline | Not  stated | No | Not stated | Not  stated | No | No | No |
| Wheatcroft S, 2003^48^ | Pathophysiological implications of insulin resistance on vascular endothelial function | | No  (Narrow) | Yes | No | No | Adequate | Medline | Yes | No | Yes | Not  stated | No | No | No |
| Wilson M, 2003^49^ | Molecular epidemiology of pre-eclampsia | | Yes  (Broad) | Yes | No | No | Adequate | Medline | Yes | No | Yes | Yes | Yes | No | No |
| Yao V, 2003^50^ | Role of peritoneal mesothelial cells in peritonitis | | Yes  (Narrow) | Yes | No | No | Adequate | Medline | Yes | No | Yes | Not  stated | No | No | No |
| Bremnes R, 2002^51^ | The E-cadherin cell-cell adhesion complex and lung cancer invasion, metastasis, and prognosis | | Yes  (Narrow) | Yes | No | No | Inadequate | Medline | Not  stated | No | Not stated | Not  stated | No | No | No |
| Fairey A, 2002^52^ | Physical Exercise and Immune System Function | | Yes  (Broad) | Yes | No | No | Inadequate | Medline | Not  stated | No | Not stated | Yes | Yes | No | No |
| Kitchens K, 2002^53^ | Contact factors in venous or arterial thromboembolism or acute coronary syndromes. | | Yes  (Broad) | Yes | No | No | Inadequate | Medline | Yes | No | Not stated | Not  stated | Yes | No | No |
| Ray E, 2002^54^ | Growth factor regulation of enterocyte nutrient transport during intestinal adaptation | | No  (Narrow) | Yes | No | No | Inadequate | Medline | Not  stated | No | Not stated | Not  stated | No | No | No |
| Reeves S, 2002^55^ | Ageing and the nigrostriatal dopaminergic system | | Yes  (Narrow) | Yes | No | No | Adequate | Medline | Yes | No | Not stated | Not  stated | Yes | No | No |
| Schellekens H, 2002^56^ | Immunogenicity of Therapeutic Proteins | | Yes  (Broad) | No | Yes | No | Adequate | Medline, Embase | No | No | Not stated | Not  stated | No | No | No |
| Sosen I, 2002^57^ | Interactions of cytokines, growth factors, and the extracellular matrix in the cellular biology of uterine leiomyomata | | Yes  (Narrow) | Yes | No | Yes | Inadequate | Medline | Not  stated | No | Not stated | Not  stated | No | No | No |
| Stanford J, 2002^58^ | Mechanisms of action of intrauterine devices: | | Yes  (Narrow) | No | Yes | No | Adequate | Medline, Popline | Yes | No | Not stated | Not  stated | Yes | No | Yes |
| Vallet B, 2002^59^ | Endothelial cell dysfunction and abnormal tissue perfusion | | Yes  (Narrow) | Yes | No | No | Inadequate | Medline | Not  stated | No | Not stated | Not  stated | No | No | No |
| Grizzle W, 2001^60^ | Molecular markers in colorectal neoplasia | | Yes  (Narrow) | Yes | No | No | Inadequate | Medline, Biomednet, | Not  stated | No | Yes | Not  stated | No | No | No |
| Harada T, 2001^61^ | Role of cytokines in endometriosis | | Yes  (Narrow) | Yes | No | No | Inadequate | Medline | yes | No | Not stated | Not  stated | No | No | No |
| Kalis M, 2001^62^ | Pharmacology and therapeutic use of oxcarbazepine | | Yes  (Broad) | No | Yes | No | Adequate | Medline, Embase, Pharmaceutical Abstracts | Not  stated | No | Not stated | Not  stated | No | No | No |
| Miller J, 2001^63^ | The biology of natural killer cells in cancer, infection, and pregnancy | | Yes  (Broad) | Yes | No | Yes | Adequate | Medline | Not  stated | No | Not stated | Not  stated | No | No | No |
| Mourits M, 2001^64^ | Tamoxifen treatment and gynecologic side effects | | Yes  (Broad) | No | Yes | No | Adequate | Medline | Not  stated | No | Not stated | Not  stated | No | No | No |
| Serracino-Inglott B, 2001^65^ | Hepatic ischemia-reperfusion injury | | No  (Narrow) | Yes | No | No | Adequate | Medline | Not  stated | No | Not stated | Not  stated | No | No | No |
| Burchardt M, 2000^66^ | Biomarker technology for bladder Cancers | | No  (Broad) | No | No | No | Adequate | Medline | Not  stated | No | Not stated | Not  stated | Yes | No | No |
| De Simone J, 2000^67^ | Inflammatory Reactions in HIV-1–Infected Persons after Initiation of Highly Active Antiretroviral Therapy | | Yes  (Narrow) | No | Yes | No | Inadequate | Medline | Yes | No | Not stated | Not  stated | No | No | No |
| Geva E, 2000^68^ | Role of vascular endothelial growth factor in ovarian physiology and pathology | | Yes  (Narrow) | Yes | No | No | Inadequate | Medline, Current Contents, and the Index Medicus | Yes | No | Yes | Not  stated | No | No | No |
| Golledge J, 2000^69^ | The Symptomatic Carotid Plaque | | No  (Narrow) | Yes | No | No | Inadequate | Medline | Not  stated | No | Not stated | Not  stated | Yes | No | No |
| Green C, 1999^70^ | Effectiveness of craniosacral interventions | | Yes  (Narrow) | No | Yes | Yes | Adequate | Medline, Embase Healthstar, Mantis, Allied, Alternaticve medicine, Scisearch, Biosis | Yes | No | Not stated | Yes | Yes | No | No |
| Ibrahim N, 1999^71^ | The evolving role of specic estrogen receptor modulators (SERMS) | | Yes  (Broad) | Yes | No | No | Inadequate | Medline, Cancerlit | Not  stated | No | Not stated | Not  stated | Yes | No | No |
| Samprieto M, 1999^72^ | Molecular pathology of Crigler-Najjar type I and II and Gilbert’s syndromes | | Yes  (Broad) | Yes | No | No | Inadequate | Medline, Science Citation index | Not  stated | No | Not stated | Not  stated | No | No | No |
| Faura C, 1998^73^ | Factors affecting the ratios of morphine and its major metabolites | | Yes  (Narrow) | Yes | No | Yes | Adequate | Embase, Medline Relief Database, Biological  Abstracts | Yes | No | Not stated | Yes | Yes | No | No |
| ter Riet G, 1998^74^ | Is placebo analgesia mediated by endogenous opioids? | | Yes  (Narrow) | No | Yes | Yes | Inadequate | Medline, Embase, Psyclit, Cochrane Library, Science Citation Index | Not  stated | No | Not stated | Yes | Yes | No | No |
| Looney S,1997^75^ | Sodium, potassium-activated adenosine triphosphatase in bipolar illness | | Yes  (Narrow) | Yes | No | Yes | Inadequate | Index Medicus | yes | Yes | Not stated | Yes | Yes | Yes | Yes |

**Appendix 1 References**

1. Borrelli F, Izzo AA, Ernst E. Pharmacological effects of Cimicifuga racemosa. *Life Sciences.* 2003;**73**:1215-29.

2. Bailey B. Glucagon in beta-blocker and calcium channel blocker overdoses: A systematic review. *Journal of Toxicology - Clinical Toxicology.* 2003;**41**:595-602.

3. Corpet DE,.Tache S. Most effective colon cancer chemopreventive agents in rats: a systematic review of aberrant crypt foci and tumor data, ranked by potency. *Nutr Cancer* 2002;**43**:1-21.

4. Dirx MJ, Zeegers MP, Dagnelie PC, Van Den BT, Van Den Brandt PA. Energy restriction and the risk of spontaneous mammary tumors in mice: a meta-analysis. *International Journal of Cancer.* 2003;**106**:766-70.

5. Holemans K, Aerts L, Van Assche FA. Fetal growth restriction and consequences for the offspring in animal models. *J Soc Gynecol Investig.* 2003;**10**:392-9.

6. Lee DS, Nguyen QT, Lapointe N, Austin PC, Ohlsson A, Tu JV *et al*. Meta-analysis of the effects of endothelin receptor blockade on survival in experimental heart failure. *J Card Fail.* 2003;**9**:368-74.

7. Mapstone J, Roberts I, Evans P. Fluid resuscitation strategies: a systematic review of animal trials. *Journal of Trauma-Injury Infection & Critical Care.* 2003;**55**:571-89.

8. Meune C, Spaulding C, Mahe I, Lebon P, Bergmann J-F. Risks versus Benefits of NSAIDs Including Aspirin in Myocarditis: A Review of the Evidence from Animal Studies. *Drug Safety.* 2003;**26**:975-81.

9. Quadrilatero J,.Hoffman-Goetz L. Physical activity and colon cancer. A systematic review of potential mechanisms. *J Sports Med Phys.Fitness* 2003;**43**:121-38.

10. Rehman J, Landman J, Sundaram C, Clayman RV. Tissue chemoablation. *J Endourol.* 2003;**17**:647-57.

11. Ren Y, Maltha JC, Kuijpers-Jagtman AM. Optimum force magnitude for orthodontic tooth movement: a systematic literature review. *Angle Orthodontist.* 2003;**73**:86-92.

12. Aghajafari F, Murphy K, Matthews S, Ohlsson A, Amankwah K, Hannah M. Repeated doses of antenatal corticosteroids in animals: A systematic review. *American Journal of Obstetrics & Gynecology.* 2002;**186**:843-9.

13. Fox CH,.Eberl M. Phytic acid (IP6), novel broad spectrum anti-neoplastic agent: A systematic review. *Complementary Therapies in Medicine.* 2002;**10**:229-34.

14. Kilborn SH, Trudel G, Uhthoff H. Review of growth plate closure compared with age at sexual maturity and lifespan in laboratory animals. *Contemporary Topics in Laboratory Animal Science.* 2002;**41**:21-6.

15. Klinge B, Gustafsson A, Berglundh T. A systematic review of the effect of anti-infective therapy in the treatment of peri-implantitis. *J Clin Periodontol.* 2002;**29 Suppl 3**:213-25.

16. Lucas C, Criens-Poublon LJ, Cockrell CT, de Haan RJ. Wound healing in cell studies and animal model experiments by Low Level Laser Therapy; were clinical studies justified? a systematic review. *Lasers Med Sci.* 2002;**17**:110-34.

17. Blackman K, Brown SG, Wilkes GJ. Plasma alkalinization for tricyclic antidepressant toxicity: a systematic review. *Emerg.Med (Fremantle)* 2001;**13**:204-10.

18. Boissier J,.Mone H. Relationship between worm burden and male proportion in Schistosoma mansoni experimentally infected rodents and primates. A meta-analytical approach. *Int.J Parasitol.* 2001;**31**:1597-9.

19. Collins JJ, Ness R, Tyl RW, Krivanek N, Esmen NA, Hall TA. A review of adverse pregnancy outcomes and formaldehyde exposure in human and animal studies. *Regulatory Toxicology & Pharmacology.* 2001;**34**:17-34.

20. Horn J, de Haan RJ, Vermeulen M, Luiten PG, Limburg M. Nimodipine in animal model experiments of focal cerebral ischemia: a systematic review. *Stroke* 2001;**32**:2433-8.

21. Rantala H, Tarkka R, Uhari M. Systematic review of the role of prostaglandins and their synthetase inhibitors with respect to febrile seizures. *Epilepsy Res* 2001;**46**:251-7.

22. Sherer DM,.Abulafia O. Angiogenesis during implantation, and placental and early embryonic development. *Placenta* 2001;**22**:1-13.

23. Fishbain DA, Cutler R, Rosomoff HL, Rosomoff RS. Evidence-based data from animal and human experimental studies on pain relief with antidepressants: a structured review. *Pain Med* 2000;**1**:310-6.

24. Glatt SJ, Bolanos CA, Trksak GH, Jackson D. Effects of prenatal cocaine exposure on dopamine system development: a meta-analysis. *Neurotoxicology & Teratology.* 2000;**22**:617-29.

25. Nava-Ocampo AA, Reyes-Perez H, Bello-Ramirez AM, Mansilla-Olivares A, Ponce-Monter H. For ischemic brain damage, is preclinical evidence of neuroprotection by presynaptic blockade of glutamate release enough? *Medical Hypotheses.* 2000;**54**:77-9.

26. Abulafia O,.Sherer DM. Angiogenesis of the endometrium. *Obstet Gynecol* 1999;**94**:148-53.

27. Vickers AJ. Independent replication of pre-clinical research in homeopathy: a systematic review. *Forsch.Komplementarmed.* 1999;**6**:311-20.

28. Kelley G. Mechanical overload and skeletal muscle fiber hyperplasia: A meta- analysis. *Journal of Applied Physiology.Vol.81(4)()(pp 1584-1588), 1996.* 1996;1584-8.

29. Laviano A, Renvyle T, Yang ZJ. From laboratory to bedside: new strategies in the treatment of malnutrition in cancer patients. *Nutrition* 1996;**12**:112-22.

30. Perna A,.Remuzzi G. Abnormal permeability to proteins and glomerular lesions: a meta-analysis of experimental and human studies. *Am J Kidney Dis.* 1996;**27**:34-41.

31. Abraham E. Neutrophils and acute lung injury. *Critical Care Medicine.Vol.31(4 SUPPL.)()(pp S195-S199), 2003.Date of Publication: 01 APR 2003.* 2003;S195-S199.

32. Alam M, Hsu T-S, Dover JS, Wrone DA, Arndt KA. Nonablative laser and light treatments: Histology and tissue effects - A review. *Lasers in Surgery & Medicine.* 2003;**33**:30-9.

33. Al Sukhun S,.Hussain M. Current understanding of the Biology of advanced bladder cancer. *Cancer.* 2003;**97**:2064-75.

34. Bellanti JA, Malka-Rais J, Castro HJ, Mendez d, I, Sabra A. Developmental immunology: Clinical application to allergy-immunology. *Annals of Allergy, Asthma, & Immunology.* 2003;**90**:2-6.

35. Creteur J,.Vincent J-L. Hemoglobin solutions. *Critical Care Medicine* 2003;**13**:S698-S707.

36. Debattista J, Timms P, Allan J, Allan J. Immunopathogenesis of Chlamydia trachomatis infections in women. *Fertility & Sterility.* 2003;**79**:1273-87.

37. Duggan BJ, McKnight JJ, Williamson KE, Loughrey M, O'Rourke D, Hamilton PW *et al*. The need to embrace molecular profiling of tumor cells in prostate and bladder cancer. *Clinical Cancer Research.* 2003;**9**:1240-7.

38. Fenster BE, Tsao PS, Rockson SG. Endothelial dysfunction: Clinical strategies for treating oxidant stress. *American Heart Journal* 2003;**146**:218-26.

39. Garcea G, Dennison AR, Steward WP, Berry DP. Chemoprevention of gastrointestinal malignancies. *ANZ Journal of Surgery.* 2003;**73**:680-6.

40. Kanayama N. Trophoblastic injury: New etiological and pathological concept of preeclampsia. *Croatian Medical Journal.Vol.44(2)()(pp 148-156), 2003.* 2003;148-56.

41. Koornstra JJ, De Jong S, Hollema H, De Vries EGE, Kleibeuker JH. Changes in apoptosis during the development of colorectal cancer: A systematic review of the literature. *Critical Reviews in Oncology-Hematology.* 2003;**45**:37-53.

42. Lyall M, Peakman M, Wessely S. A systematic review and critical evaluation of the immunology of chronic fatigue syndrome. *Journal of Psychosomatic Research.* 2003;**55**:79-90.

43. Mendez JD. Advanced glycation end product and chronic complications of diabetes mellitus. *Gaceta Medica de Mexico.* 2003;**139**:49-55.

44. Norbury R, Cutter WJ, Compton J, Robertson DM, Craig M, Whitehead M *et al*. The neuroprotective effects of estrogen on the aging brain. *Experimental Gerontology.* 2003;**38**:109-17.

45. Poon RTP, Fan S-T, Wong J. Clinical Significance of Angiogenesis in Gastrointestinal Cancers: A Target for Novel Prognostic and Therapeutic Approaches. *Annals of Surgery.* 2003;**238**:9-28.

46. Reijnen MMPJ, Bleichrodt RP, Van Goor H. Pathophysiology of intra-abdominal adhesion and abscess formation, and the effect of hyaluronan. *British Journal of Surgery.* 2003;**90**:533-41.

47. Szabo C. Role of flagellin in the pathogenesis of shock and acute respiratory distress syndrome: Therapeutic opportunities. *Critical Care Medicine.* 2003;**31**:S39-S45.

48. Wheatcroft SB, Williams IL, Shah AM, Kearney MT. Pathophysiological implications of insulin resistance on vascular endothelial function. *Diabetic Medicine.* 2003;**20**:255-68.

49. Wilson ML, Goodwin TM, Pan VL, Ingles SA. Molecular epidemiology of preeclampsia. *Obstetrical & Gynecological Survey.Vol.58(1)()(pp 39-66), 2003.Date of Publication: 01 JAN 2003.* 2003;39-66.

50. Yao V, Platell C, Hall JC. Role of peritoneal mesothelial cells in peritonitis. *British Journal of Surgery.* 2003;**90**:1187-94.

51. Bremnes RM, Veve R, Hirsch FR, Franklin WA. The E-cadherin cell-cell adhesion complex and lung cancer invasion, metastasis, and prognosis. *Lung Cancer.* 2002;**36**:115-24.

52. Fairey AS, Courneya KS, Field CJ, Mackey JR. Physical exercise and immune system function in cancer survivors: A comprehensive review and future directions. *Cancer.* 2002;**94**:539-51.

53. Kitchens CS. The contact system. *Archives of Pathology & Laboratory Medicine.* 2002;**126**:1382-6.

54. Ray EC, Avissar NE, Sax HC. Growth factor regulation of enterocyte nutrient transport during intestinal adaptation. *American Journal of Surgery.* 2002;**183**:361-71.

55. Reeves S, Bench C, Howard R. Ageing and the nigrostriatal dopaminergic system. *International Journal of Geriatric Psychiatry.* 2002;**17**:359-70.

56. Schellekens H. Immunogenicity of therapeutic proteins: clinical implications and future prospects. *Clinical Therapeutics.* 2002;**24**:1720-40.

57. Sozen I,.Arici A. Interactions of cytokines, growth factors, and the extracellular matrix in the cellular biology of uterine leiomyomata. *Fertility & Sterility.* 2002;**78**:1-12.

58. Stanford JB,.Mikolajczyk RT. Mechanisms of action of intrauterine devices: update and estimation of postfertilization effects. *American Journal of Obstetrics & Gynecology.* 2002;**187**:1699-708.

59. Vallet B. Endothelial cell dysfunction and abnormal tissue perfusion. *Critical Care Medicine.* 2002;**30**:S229-S234.

60. Grizzle WE, Manne U, Jhala NC, Weiss HL. Molecular characterization of colorectal neoplasia in translational research. *Archives of Pathology & Laboratory Medicine.* 2001;**125**:91-8.

61. Harada T, Iwabe T, Terakawa N. Role of cytokines in endometriosis. *Fertility & Sterility.Vol.76(1)()(pp 1-10), 2001.* 2001;1-10.

62. Kalis MM,.Huff NA. Oxcarbazepine, an antiepileptic agent. *Clinical Therapeutics.* 2001;**23**:680-700.

63. Miller JS. The biology of natural killer cells in cancer, infection, and pregnancy. *Experimental Hematology.Vol.29(10)()(pp 1157-1168), 2001.* 2001;1157-68.

64. Mourits MJE, De Vries EGE, Willemse PHB, Ten Hoor KA, Hollema H, Van der Zee AGJ. Tamoxifen treatment and gynecologic side effects: A review. *Obstetrics & Gynecology.Vol.97(5)()(pp 855-866), 2001.* 2001;855-66.

65. Serracino-Inglott F, Habib NA, Mathie RT. Hepatic ischemia-reperfusion injury. *American Journal of Surgery.Vol.181(2)()(pp 160-166), 2001.* 2001;160-6.

66. Burchardt M, Burchardt T, Shabsigh A, De la TA, Benson MC, Sawczuk I. Current concepts in biomarker technology for bladder cancers. *Clinical Chemistry.Vol.46(5)()(pp 595-605), 2000.* 2000;595-605.

67. DeSimone JA, Pomerantz RJ, Babinchak TJ. Inflammatory reactions in HIV-1-infected persons after initiation of highly active antiretroviral therapy. *Annals of Internal Medicine.* 2000;**133**:447-54.

68. Geva E, Amit A, Lerner-Geva L, Lessing JB. Autoimmunity and reproduction. *Fertility & Sterility.* 1997;**67**:599-611.

69. Golledge J, Greenhalgh RM, Davies AH. The symptomatic carotid plaque. *Stroke.* 2000;**31**:774-81.

70. Green C, Martin CW, Bassett K, Kazanjian A. A systematic review of craniosacral therapy: biological plausibility, assessment reliability and clinical effectiveness. *Complement Ther.Med* 1999;**7**:201-7.

71. Ibrahim NK,.Hortobagyi GN. The evolving role of specific estrogen receptor modulators (SERMs). *Surgical Oncology.Vol.8(2)()(pp 103-123), 1999.* 1999;103-23.

72. Sampietro M,.Iolascon A. Molecular pathology of Crigler-Najjar type I and II and Gilbert's syndromes. *Haematologica.Vol.84(2)()(pp 150-157), 1999.* 1999;150-7.

73. Faura CC, Collins SL, Moore RA, McQuay HJ. Systematic review of factors affecting the ratios of morphine and its major metabolites. *Pain* 1998;**74**:43-53.

74. ter Riet G, de Craen AJ, de Boer A, Kessels AG. Is placebo analgesia mediated by endogenous opioids? A systematic review. *Pain* 1998;**76**:273-5.

75. Looney SW,.El Mallakh RS. Meta-alnaysis of erythrocyte Na,K-ATPase activity in bipolar illness. *Depression & Anxiety.Vol.5(2)()(pp 53-65), 1997.* 1997;53-65.
